# Survey on the Application of Robotics in Archaeology

**DOI:** 10.3390/s25154836

**Published:** 2025-08-06

**Authors:** Panagiota Kyriakoulia, Anastasios Kazolias, Dimitrios Konidaris, Panagiotis Kokkinos

**Affiliations:** 1 Department of Digital Systems, University of Peloponnese, 22100 Tripoli, Greece; bettykyriakoulia10@gmail.com (P.K.); tkazolias1992@gmail.com (A.K.); dkonidaris@go.uop.gr (D.K.); 2 Institute of Communication and Computer Systems (ICCS), National Technical University of Athens, 15772 Zografou, Greece

**Keywords:** robotics, archaeology, cultural heritage, AUV, UAV, ROV, LiDAR, 3D modeling, use cases

## Abstract

This work explores the application of robotic systems in archaeology, highlighting their transformative role in excavation, documentation, and the preservation of cultural heritage. By combining technologies such as LiDAR, GIS, 3D modeling, sonar, and other sensors with autonomous and semi-autonomous platforms, archaeologists can now reach inaccessible sites, automate artifact analysis, and reconstruct fragmented remains with greater precision. The study provides a systematic overview of underwater, aerial, terrestrial, and other robotic systems, drawing on scientific literature that showcases their innovative use in both fieldwork and museum settings. Selected examples illustrate how robotics is being applied to solve key archaeological challenges in new and effective ways. While the paper emphasizes the potential of these technologies, it also addresses their technical, economic, and ethical limitations, concluding that successful adoption depends on interdisciplinary collaboration, careful implementation, and a balanced respect for cultural integrity.

## 1. Introduction

Archaeology is the science that investigates and studies the culture of people through the material remains and structures of the past, constituting one of the most fascinating fields of research. The preservation and study of cultural heritage are among humanity’s most significant tasks, as they enable future generations to comprehend their historical identity and transmit knowledge of the past to the future. The relationship between modern individuals and archaeology is close and undeniable, as it serves as a primary source of information about the past and people’s way of life and achievements, helping us understand the evolution that has taken place over time and the events of the past [1]. Archaeology is a multifaceted science, and it is difficult to fully define with a single definition, as its goals and functions continuously evolve over time. A general definition is that archaeology is the humanistic science that aims to reconstruct and understand near or distant ancient human life by studying the material remains of human activity discovered during excavations [2,3]. According to [4], archaeology seeks to interpret the conditions under which people acted and lived in the past, both in local and broader historical societies, and promote historical knowledge and empathy.

In recent decades, significant technological advancements have offered new possibilities and innovative solutions, enhancing research accessibility and efficiency, with robotics occupying a central position in archaeological investigations. As a rapidly developing branch of science, it contributes to a new era in archaeological science by supporting and evolving archaeological practices. The term ’robotics’ refers to the branch of technology that focuses on the conception, design, development, and subsequent operation of a robot. It is a modern field of technology that studies the design and execution of the tasks performed by a robot and conducts research for its further development. Robotics integrates a variety of sciences, primarily computer science, mechanical engineering, and electronics, which collaborate to create a new synthetic science that deals with the exchange of information and energy between a machine and the environment in which it operates, aiming toward a specific set of objectives [5].

The connection between robotics and archaeology is not only a technological innovation but also an attempt to overcome the limitations of traditional methods, as robots are used in various tasks, such as exploring inaccessible areas; mapping underwater, subterranean, or challenging archaeological sites; and collecting data with accuracy and speed. New possibilities provided by robotics, such as 3D scanning, autonomous vehicles, photogrammetry, and augmented reality (AR), can usher in a new era for archaeology and lead to unprecedented levels of documenting cultural achievements.

The methodology followed in this study is based on a combinatorial approach, ensuring a multidimensional analysis of the subject and projecting a comprehensive view of robotics’ contribution to the science of archaeology. It includes the following components:

- Literature Review: The primary source of scientific material examined in this study is the collection and study of articles published in the press and online, as well as scientific books that present the applications of robotics both in archaeological investigation and in the museum space. The goal was not to include all possible articles found, but to provide a good understanding of this multi-disciplinary area.
- Case Studies Analysis: For this study, a number of characteristic case studies are examined to highlight the significance of using robots in archaeological research. The selection of these specific cases was made based on the innovative use of robotic technology in each case, solving archaeological issues in a pioneering way, and the importance of each robotic system in archaeological research.

Although robotic technologies are becoming more common in archaeological projects, a systematic, technology-focused review of their use in excavation, conservation, and interpretation is still lacking. Many existing reviews either focus on technological advances without connecting them to archaeological practice or discuss heritage science while overlooking the role of robotics as a key enabler. This study aims to fill this gap by providing a structured overview of how different types of robotic systems—such as underwater robots, robotic arms, aerial drones, and ground-based platforms—are reshaping archaeological workflows and methodologies. It highlights the roles these technologies play, the challenges they help address, their problem-solving potential, and the opportunities they create for cross-disciplinary collaboration. By combining classification frameworks, real-world applications, practical case studies, and ethical considerations, this review offers a valuable resource for both archaeologists and researchers working in robotics.

This article is structured as follows. Section 1 introduces the intersection of robotics and archaeology, outlining the study’s objectives and methodology. Section 2 examines key technologies (LiDAR, GIS, 3D modeling) and robotic systems (aerial, underwater, ground-based) used in archaeological research. Section 3 details applications, including site mapping, artifact restoration, and museum engagement. Section 4 presents case studies, such as the robotic restoration at Pompeii. Finally, Section 5 discusses technological, economic, and ethical challenges in the field.

## 2. Archaeology and Technology

Archaeology, as a science, has traditionally been associated with physical research, as its primary tool is excavation, through which material remnants are uncovered, aiding in the study of human civilization. The way archaeologists today discover, record, and interpret findings has shown remarkable improvement, as technology has contributed to the advancement of archaeological science. In many cases, traditional methods are still employed, such as using a journal for daily documentation of excavation processes and findings, manual drawing, and manual excavation techniques by specialized workers. Although these methods remain of great importance and have been the foundation of excavation research for centuries, they have several disadvantages, such as being time-consuming, costly, and lacking the precision provided by modern tools and the latest technological methods.

The contribution of technology to the development and evolution of archaeology is truly remarkable, as its introduction into this field has facilitated improvements and solutions to various problems arising from the exclusive use of traditional methods. Archaeology and technology, converging at excavation sites, work together in recording, preserving, and analyzing archaeological findings and data, ultimately leading to their interpretation.

### 2.1. Technologies

**Figure 1:**
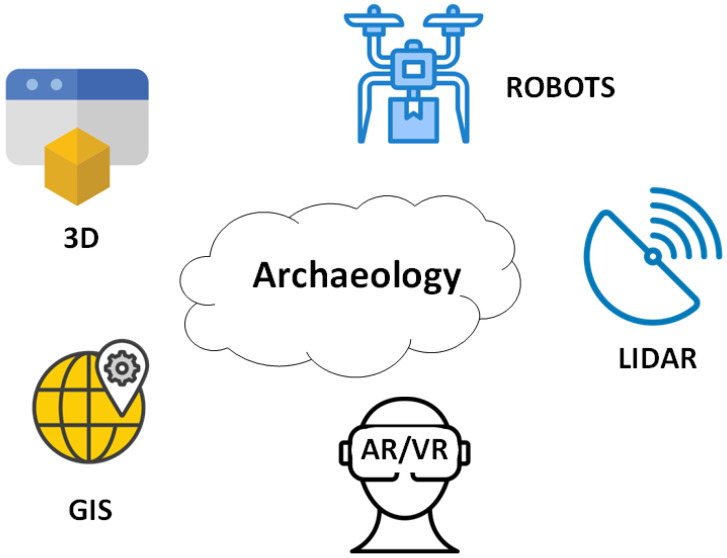
Technologies used in archaeology.

More specifically, the cutting-edge technologies applied in archaeology today include (Figure 1):

- **LiDAR (Light Detection and Ranging) Technology:** Initially used in meteorology [6], LiDAR has brought significant changes to archaeological research over the past two decades by enabling high-speed topographic mapping. It is a sensor that measures variations in the ground and creates three-dimensional maps, identifying archaeological sites that would otherwise remain undetected [7].
- **Geographic Information Systems (GIS):** GIS are digital tools that allow the identification of archaeological sites through the statistical analysis of digital images combined with archaeological and environmental information [8].
- **Three-Dimensional Modeling (3D Modeling):** This is an advanced form of digitization aimed at the three-dimensional documentation of archaeological sites and objects. The most widely used method is laser scanning, but depending on the case and the desired outcome, other techniques such as shape from structured light, shape from stereo imaging, shape from photometry, photogrammetry, and field laser scanning are also employed [9,10]. The benefits of 3D modeling in cultural heritage are numerous, including digital documentation of archaeological findings, public access to 3D archaeological objects via the internet, and the creation of accurate replicas for educational purposes or for conservation, aiding in the restoration and reconstruction of broken fragments [11].
  *Terminological Clarification: Photogrammetry vs. 3D Modeling*
  To ensure clarity and consistency in the use of technical terminology, it is important to distinguish between **photogrammetry** and **3D modeling**, which are sometimes used interchangeably in archaeological literature.
  - **Photogrammetry** refers to the process of capturing a sequence of overlapping images of an object or site from multiple angles and using computer algorithms to extract geometric information. This method enables the generation of accurate spatial measurements and visual representations.
  - **Three-dimensional modeling**, by contrast, refers to the digital reconstruction or representation of objects or environments in three dimensions. It can be produced using various data acquisition techniques, including photogrammetry, laser scanning, and structured light scanning.
  In other words, photogrammetry is a *data acquisition method*, while 3D modeling is the *resulting output* that may be generated through different techniques. Throughout this article, these terms are used with technical precision to avoid confusion and ensure terminological accuracy [9,12].
- **Augmented Reality (AR) and Virtual Reality (VR):** AR and VR technologies enable immersive visualization and interaction with archaeological sites and artifacts. These tools allow researchers to reconstruct and explore ancient structures in three dimensions, providing virtual tours and interpretative layers that enhance both academic research and public engagement.
  *Terminological Clarification: AR vs. VR*
  Although these are often mentioned together, augmented reality and virtual reality refer to distinct technologies with different use cases in archaeological projects:
  - **Augmented Reality (AR)** overlays digital content—such as text, images, or 3D models—onto the real world through a smartphone, tablet, or AR headset. It enables on-site enhancement by contextualizing physical ruins with reconstructions or annotations [13,14].
  - **Virtual Reality (VR)** creates an entirely simulated digital environment, immersing the user in a 3D reconstruction of an archaeological site or artifact. VR is often used for virtual site tours, remote access to inaccessible excavations, and educational applications [15].
  The use of AR and VR has transformed how cultural heritage is documented, studied, and communicated. These technologies support digital storytelling, hypothesis testing, and public dissemination of archaeological knowledge, and they are increasingly integrated into museum exhibitions, educational platforms, and preservation strategies [16].

### 2.2. Categories of Robots

As previously mentioned, robots are devices designed to perform preprogrammed tasks and perceive their environment, and they can be categorized as follows:

- **Fixed-base robots**: This type consists of links—solid bodies that form a kinematic chain. One end is attached to a fixed base in space, which connects to the other links through joints. These joints enable movement and can be classified based on their degrees of freedom as rotary, prismatic, or spherical.
- **Mobile Robots**: These robots can move in space using wheels, propellers, rotors, or mechanical legs. Mobile robots are further classified based on their mode of movement and degree of autonomy:
  - **Wheeled Autonomous Robots**: These robots move using wheels and possess a high degree of autonomy. They do not require continuous supervision and are capable of executing high-level commands.
  - **Legged Robots**: These robots use mechanical legs for movement. Unlike wheeled robots, they can more easily navigate uneven terrain and overcome obstacles.
  - **Aerial Robots (UAVs and Drones)**: These are flying, unmanned robots capable of continuous flight and performing predefined tasks without direct operator control. They may operate autonomously or be remotely controlled from the ground. In recent years, significant research progress has been made in this area.
- **Remotely Operated Vehicles (ROVs) and Autonomous Underwater Vehicles (AUVs)**: These are unmanned underwater robots used in industries such as oil, gas, and mineral exploration, as well as subsea geotechnical surveys [17]. They are known for their flexibility, with sizes ranging from small observation units to large systems capable of complex operations. Advantages of ROVs include unlimited operational time (since they are powered by a surface vessel), the ability to access areas unsafe for divers, and detailed seabed inspections. However, limitations include restricted movement due to the tether cable, challenges operating in strong currents, and difficulty in very shallow waters. An AUV operates independently without a tether, following pre-programmed missions for tasks like mapping or surveys.
  In archaeological practice, ROVs and AUVs have become crucial tools for underwater exploration and, more particularly, in the documentation and analysis of shipwrecks and submerged sites. As part of the ARROWS project, ROVs carrying advanced imaging technologies such as high-resolution cameras and sonar were deployed to perform non-invasive mapping of submerged archaeological remains across the Mediterranean Sea. Such robotic systems facilitate high-precision 3D modeling of submerged heritage sites, particularly in environments where it is unsafe or unfeasible for divers to have access. Additionally, AUVs have been utilized in self-guided survey operations, like that at the Kolumbo submarine volcano near Santorini (Greece), where they produced high-resolution bathymetric maps and contributed to the detection of archaeological features. These robotic systems not only increase operational safety and efficiency in underwater exploration but also play a basic role in long-term preservation by reducing direct human interference with sensitive marine ecosystems [18,19].
- **Unmanned Ground Robots (UGVs)**: These robots play an increasingly important role in archaeological explorations where conditions or site fragility prevent safe human entry. They are especially effective in navigating collapsed structures, unstable ruins, or confined underground environments. For instance, quadruped robots such as Spot have been used at Pompeii to inspect tunnels and monitor the integrity of ancient buildings without risking structural damage from human activity. Furthermore, UGVs can be equipped with ground-penetrating radar (GPR) to assist in the non-invasive detection of buried artifacts and structures, offering archaeologists critical insights before digging begins [20].
- **Manipulative Robots**: In archaeological conservation, manipulative robots are gaining ground in the automated handling, classification, and reassembly of fragmented artifacts. Systems like RASCAL and the EU-funded RePAIR project (Reconstructing the Past: Artificial Intelligence and Robotics meet Cultural Heritage) have shown that robotic arms equipped with computer vision and AI matching algorithms can accelerate and enhance the process of reconstructing pottery, frescoes, and other cultural materials. These robots reduce the risk of human error or any damage during artifact manipulation and offer repeatable precision, which is crucial when dealing with fragile or unique objects.

To offer a better and clearer comparative perspective, Table 1 provides a well-structured overview of the main categories of robots, their core functions, technologies, and archaeological applications.

## 3. Applications of Robotics in Archaeology

**Figure 2:**
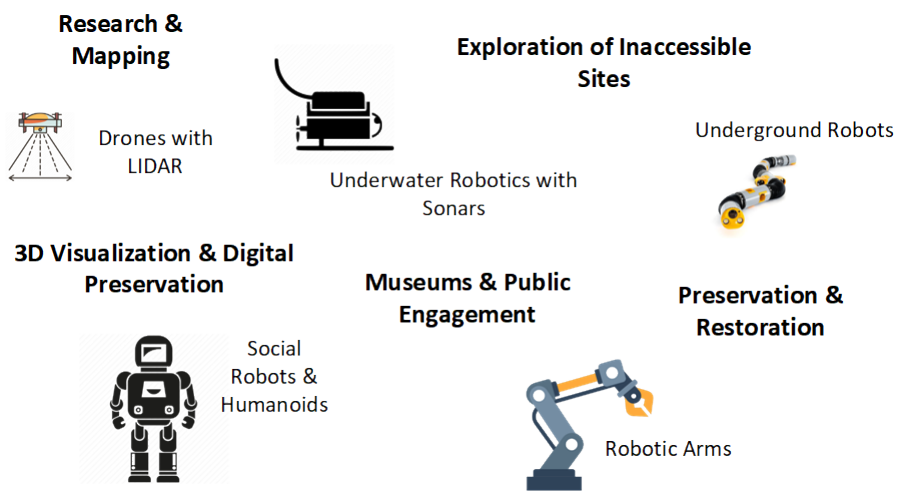
Robots in archaeology.

An emerging interdisciplinary field at the intersection of archaeology and robotics has gained significant attention in recent years, aiming to explore, study, and preserve cultural monuments and archaeological findings uncovered through excavations (Figure 2). This important collaboration between the humanities and engineering enables the adaptation of technological solutions to the specific needs of archaeological research and fieldwork while also contributing to the preservation and sustainable management of cultural heritage [27]. Robotics applications, in particular, have introduced new capabilities for mapping, visualization, and in-depth analysis of archaeological sites. Notable innovations include the use of drones and 3D imaging technologies, which—both individually and in combination—support the creation of interactive tools and promote a deeper, broader understanding of cultural heritage.

New technologies are being developed and applied within a dynamic framework that brings together novel theoretical approaches with technical knowledge and innovation. This synergy promotes the sustainable management of cultural heritage.

### 3.1. Research and Mapping

Aerial robotics has become a key asset in archaeological survey work, enabling the efficient mapping of vast or inaccessible sites. These systems are frequently utilized for documenting excavation areas, supporting landscape-scale archaeological studies, and locating buried features through vegetation-based indicators. When equipped with LiDAR or multispectral sensors, UAVs can perform detailed, non-destructive mapping of topography and sub-canopy structures, support unobtrusive surveying of landforms, and detect hidden features beneath vegetation without excavation.

Unmanned aerial vehicles (UAVs), commonly known as drones, have become an essential tool in archaeology, offering topographic mapping and aerial photography capabilities for archaeological sites. Mapping an archaeological area is typically costly and time-consuming; however, drones equipped with high-resolution cameras and sensors (such as LiDAR, multispectral cameras, and other detection equipment) can collect data that enable detailed documentation of cultural heritage [28,29]. Drones provide a variety of advantages, as detailed below.

#### 3.1.1. Fast and Cost-Effective Mapping

In terms of speed and cost-efficiency, drones can rapidly collect data while significantly reducing the expenses associated with such tasks. Traditional aerial photography methods require the use of airplanes or satellites, which provide high-resolution imagery at a high cost and often with long wait times. In contrast, drones can map large archaeological areas in a short amount of time and at a cost-effective scale, making them suitable even for limited budgets [30,31].

#### 3.1.2. Access to Remote Areas

Drones offer accessibility to remote and difficult-to-reach locations where ground methods are impractical [28]. Many excavations occur in areas with challenging access, rugged terrain, dense vegetation, or even hazards, and numerous archaeological sites are located in geographically demanding environments, such as deserts or mountainous regions [32]. The ability of drones to fly at low altitudes and collect data from multiple angles provides a unique opportunity to monitor archaeological sites that are otherwise inaccessible using traditional methods [33]. Furthermore, in cases of natural disasters such as earthquakes or floods, drones can reach hazardous or obstructed areas to document the condition of archaeological monuments without putting researchers at risk [28].

#### 3.1.3. Photogrammetry and Digital Models

One revolutionary application of drones in archaeology is the creation of digital models via photogrammetry, enabling new approaches in recording and analyzing archaeological sites [12]. Photogrammetry involves capturing a series of images from different angles, which are then processed with specialized software to produce high-resolution and detailed 3D digital models. These models help in studying site geometry and monitoring wear over time, accurately documenting both ancient structures and their surrounding environment.

#### 3.1.4. Combining Drones and LiDAR

Field surveys and archaeological excavations are crucial for uncovering findings that, once interpreted, can provide significant insights into historical contexts. In recent years, modern methods have been introduced to address the limitations of past approaches in surveying even the most inaccessible areas. Accurate topographic diagrams and precise recording of movable artifacts during excavations are critical for identifying their differences and contextual relationships [34,35].

Mobile mapping systems have revolutionized archaeological site documentation by collecting high-precision data, capturing large-scale details, and producing 3D models while reducing the time and cost of traditional geospatial measurements [36]. Advancements in ground-based surveys now allow for the accurate collection of large datasets through UAV technology and the integration of LiDAR (light detection and ranging).

LiDAR emits laser pulses that detect objects and simultaneously measure their distance from the sensor. Drones, due to their compact size and mobility, are easily deployable and can scan ground surfaces equipped with LiDAR. The laser pulses reflect off surfaces, return to the sensor, and are recorded as point data [37]. When combined with GIS (geographic information systems), LiDAR point clouds are processed to analyze and manage large geospatial datasets, aiding in pattern recognition and spatial modeling and creating high-value maps [38,39]. LiDAR can reveal what optical cameras cannot: hidden archaeological structures beneath vegetation or the earth’s surface [28]. This non-invasive technique uncovers archaeological sites and features that would otherwise remain invisible using traditional methods [40]. To conduct a LiDAR-equipped drone survey, post-processing data analysis and visualization is required [41].

### 3.2. Exploring Robotics in Archaeology

Robotic technology is one of the most valuable tools in archaeology, as it aids in the exploration of areas that would typically be difficult or impossible for scientists and researchers to access. These areas include caves, underground tombs, narrow tunnels, and sites that have suffered from collapse. Robots can gather data with the highest accuracy, increasing the efficiency of archaeological research, reducing risks to researchers, and providing useful information without disturbing the surrounding environment [42,43].

#### 3.2.1. Robots for Underground Excavations

A new and innovative approach to exploring underground excavations involves the use of robots, which offers several advantages compared to traditional methods [44]. Robots used in subterranean structures are equipped with advanced technologies such as acoustic sensors, 3D imaging, and LiDAR, enabling precise mapping of spaces [45]. Specifically, LiDAR allows for the accurate reconstruction of 3D models of areas, even in low-light environments with insufficient natural illumination [37]. Reference [46] presents the development of a compact, articulated hexapod robot named A-RHex designed to assist archaeologists in the initial exploration of underground tombs. Its small size and articulated design enable navigation through narrow and complex terrains, facilitating safe and efficient pre-exploration of archaeological sites.

#### 3.2.2. Penetration of Narrow Passages and Caves

A challenging aspect of archaeological research is the exploration of narrow passages, caves, and generally inaccessible areas, where the use of robotics represents a truly innovative approach [47]. The rapid development of robotics provides safe access to narrow pathways, unstable rocks, and areas with limited visibility that are impossible for researchers to visit. Furthermore, the use of micro-robots or coordinated swarms of robots [48] opens up new possibilities for analysis in extremely tight spaces and irregular surfaces, mapping and analyzing areas where prehistoric wall paintings and other high-archaeological significance findings may exist, with minimal environmental disturbance [49,50].

#### 3.2.3. Robots in Underwater Archaeology

AUVs, in contrast, operate without a physical connection to the surface [51]. Both ROVs [52,53] and AUVs [18,54,55,56,57] are widely used in underwater archaeological missions. Other works also explore *bimanual humanoid underwater robots* with articulated arms [58] and *modular AUVs* equipped with manipulation arms [59] for complex underwater tasks. Additionally, multi-AUV collaboration has been investigated in the context of marine archaeological missions [60,61,62]. Also, in [63], a biomimetic robotic fish (Robofish) was used for underwater archaeological tasks, while in [64], an autonomous surface vehicle (ASV) was employed. These robotic systems are deployed in diverse environments—from *Mediterranean shipwrecks* [58] to *deep-water surveys in Trondheim Harbour* [53] and *cistern system mapping in Malta* [54]. Also, to support their missions, these robots integrate a variety of sensors, as summarized in Table 2.

### 3.3. 3D Visualization of Archaeological Sites

AI-powered robots can detect, record, and identify components of archaeological artifacts, facilitating the creation of digital archives and 3D models [65,66]. In particular, 3D scanning and modeling technologies have been widely used to create interactive models that revolutionize documentation, study, and preservation practices of monuments, even in remote or inaccessible areas [67]. This technology involves the use of equipment such as laser scanners, drones with photogrammetry capabilities, and 3D modeling software. Digital technologies also support the creation of virtual tours, augmented reality (AR) representations, and applications that reconstruct the original appearance of monuments, contributing to both the preservation and dissemination of cultural knowledge [68]. Through these digital reconstructions, visitors can explore these sites remotely, thus increasing public access and offering a new dimension in understanding history. Furthermore, integrating such tools into museums and educational programs enhances cultural education and public awareness [69].

### 3.4. Preservation and Restoration

Robotics is increasingly being applied in the fields of preservation and restoration of fragile archaeological findings as well as the maintenance of archaeological sites. This represents an innovative and interdisciplinary field, combining both the humanities and technological sciences.

#### 3.4.1. Applications of Robotics in Conservation and Restoration

Environmental factors such as air pollution, climate change, and the passage of time accelerate the decay of heritage monuments—especially those exposed to open air. Materials of historical structures, including temples, ancient theaters, façades, and marble sculptures, are particularly vulnerable. Traditional cleaning and maintenance techniques often involve significant risk of human-induced damage. Therefore, the deployment of advanced robotic technologies and specialized systems has become a necessity, offering safer, more effective, and less invasive conservation approaches.

Robotic systems automate a wide range of tasks that enhance the efficiency and precision of artifact restoration. These systems reduce the time required for such tasks and are capable of performing delicate, specialized interventions on fragile objects, ancient frescoes, and mosaics. Human intervention in highly sensitive archaeological materials is minimized, while robotic tools offer researchers improved accuracy and control. These specialized tools can be tailored to the needs of individual artifacts [70].

For example, robotic arms equipped with sensors can analyze materials such as metals, frescoes, sculptures, and ceramics in detail without compromising their integrity, thereby reducing the risk of human error [71]. This emerging field focuses on the development of robots specifically designed for preserving sensitive surfaces on site, such as monuments, and for handling ancient artifacts from excavation areas. These robots provide safe, precise, and effective methods, minimizing the risk of further deterioration caused by manual handling. For example, Ref. [72] presents a service robot designed to assist in the analysis, surveying, and restoration of fresco paintings. The robot integrates advanced sensing and manipulation capabilities to perform delicate tasks, minimizing the risk of damage to the artwork.

Ref. [73] highlights the ROVINA project, which utilizes AI-driven robotics to digitally document and preserve cultural heritage in challenging and sensitive environments such as the catacombs of Priscilla in Rome and San Gennaro in Naples. The ROVINA system combines autonomous robotic exploration with advanced 3D mapping and environmental sensing, enabling detailed and non-invasive documentation of fragile archaeological sites that are otherwise difficult to access. This project represents a significant step forward in the integration of robotics and artificial intelligence for heritage preservation, offering scalable solutions for safeguarding vulnerable cultural assets. Ref. [74] explores the application of robotic technologies in the preservation of cultural heritage, with a specific focus on the Bini artifacts in Nigeria.

#### 3.4.2. Robotics in the Retrieval and Analysis of Archaeological Finds

One of the most significant developments in archaeological robotics is the retrieval of artifacts from inaccessible or difficult-to-reach areas. Through the use of computer vision, sensors, and cameras, robotic systems can capture and analyze sequences of images to study archaeological finds and ancient remains without the risk of physical damage.

Additionally, during excavations, archaeologists often encounter large quantities of fragments—such as pottery sherds (“ostraka”), bones, and other materials—that require substantial time and expertise to classify and document. Due to practical constraints, these items are often selectively recorded. This process can be automated using a database that aids in the recognition of fragments through image analysis from portable devices. The artifacts are then categorized by size, shape, decoration, material composition, and level of degradation. The RePAIR project (Reconstructing the Past: Artificial Intelligence and Robotics meet Cultural Heritage) [75] uses AI, robotics, and computer vision to automate the retrieval, analysis, and reconstruction of fragmented archaeological finds. It enables large-scale restoration of artifacts like vases and frescoes, making cultural heritage more accessible by drastically reducing manual effort and time.

#### 3.4.3. Safeguarding Cultural Heritage

Robotics also plays a critical role in safeguarding cultural heritage, particularly in monuments located in disaster-prone, hazardous, or hard-to-reach regions. Equipped with environmental sensors, robots can detect changes in temperature and humidity, enabling early identification of material degradation and the implementation of preventive conservation measures [76].

### 3.5. Robots in Cultural Institutions and Museums

Robotic technology is emerging as a key driver of intelligent transformation in the museum sector and, more broadly, in the domain of cultural heritage. The integration of robots into museum environments represents a genuine innovation that enhances both the accessibility and educational experience of visitors. Robots can provide guided information about exhibits, improving visitor engagement, while also supporting essential tasks related to the preservation, restoration, and protection of museum collections.

Robots capable of interacting, communicating, and collaborating with humans are often referred to as *social robots*, and their operation may be either fully or partially autonomous. In the museum context, a social robot is not merely an information transmitter guiding and informing visitors but also a medium for creating novel, interactive tour experiences that differ significantly from traditional museum methods such as labels and static visual aids. Ref. [77] presents the MuseBot project, highlighting the integration of robotics and informatics to develop strategic solutions for enhancing museum experiences. Ref. [78] discusses the integration of semantic information retrieval systems in robotic museum guides to enhance user interaction and information accessibility. Ref. [79] describes CiceRobot, a cognitive robotic system designed to guide museum visitors, enhancing their experience through interactive tours. Ref. [80] discusses the deployment of the telepresence robot “Virgil” to enhance museum accessibility and visitor engagement through remote exploration. Ref. [81] explores the integration of robotic avatars in museum settings to enhance visitor engagement and provide innovative telepresence experiences.

Robots are increasingly used in museums to enhance the visitor experience. According to [82], five key features are essential for effective integration: (1) social navigation, allowing safe movement among visitors; (2) perception, enabling the robot to interpret visitor actions; (3) speech, for age-appropriate and interactive communication; (4) gestures, enhancing non-verbal interaction; and (5) behavior generation, combining all skills for adaptive responses in cultural spaces.

The use of robots and AI in museums enhances visitor experience but also presents challenges. High costs may burden smaller institutions, and some visitors prefer human interaction over technology. Overreliance on robots can reduce staff–visitor engagement, while sensor-based systems raise privacy and data protection concerns.

### 3.6. Summary of Robotic Applications in Archaeological Contexts

To provide and present a better overview of how robotics supports archaeological projects, Table 3 summarizes important examples of robotic applications across diverse environments. These cases range from pyramid interiors and underwater volcanoes to major heritage museums and even jungle landscapes. The table highlights key functionalities and distinct advantages offered by robotic systems in situational contexts, setting the stage for the in-depth case studies presented in Section 4.

## 4. Case Studies: Applications of Robotics in Archaeology

This section presents case studies of robotic applications drawn from both international and Greek archaeology. The selected examples were chosen based on criteria such as their contribution to the understanding and protection of cultural heritage, their level of technological innovation, and their geographical diversity. Based on these criteria, a comprehensive picture can be formed regarding the usefulness of robotics in the field of archaeology.

Each case study involves an analysis of the technologies used, the outcomes achieved, the challenges encountered, and the conclusions drawn regarding the future prospects of robotic technologies. The key aspects addressed in each case are as follows:

- **Application context:** The condition of the archaeological site before the implementation of robotic tools and the challenges that were being faced.
- **Description of the technology:** The technological tools and methods employed in each case study.
- **Results of the robotic application:** The achievements and discoveries that emerged, and how they contributed to the scientific understanding of the archaeological site.
- **Challenges and limitations:** The problems encountered during the application of robotic technology and whether they have been resolved or remain unsolved.
- **Lessons learned and future impact:** The most important lessons derived from each case and their expected influence on future robotic applications in archaeology.

### 4.1. Robotic Exploration of the Great Pyramid: The Djedi Project

One of the most well-documented applications of robotics in archaeology is the Djedi Project, which aimed to explore inaccessible shafts inside the Great Pyramid of Giza. The project introduced a highly specialized robotic system designed to operate in confined spaces without damaging the structure [83].

A breakdown of the system is provided below:

- **Application context:** The robotic system explored inaccessible shafts inside the Great Pyramid of Giza.
- **Description of the technology:** The robotic system featured the following:
  - An 8 mm micro snake camera capable of detailed imaging;
  - A 360 mm drill for piercing small obstacles (e.g., stone blocks);
  - Miniature sensors for precision data acquisition.
- **Results of the robotic application:** The mission successfully captured high-resolution images and data from previously unexplored passages, revealing copper handles and unknown symbols. These findings offered new insights into the pyramid’s internal architecture.
- **Challenges and limitations:** The robot was designed to operate in extremely confined environments without damaging the structure.
- **Lessons learned and future impact:** The Djedi robot is often cited as a benchmark example for robotic miniaturization and non-invasive exploration in archaeology [42]

### 4.2. The Use of Robots at the Archaeological Site of Pompeii

The ancient Roman city of Pompeii, a UNESCO World Heritage Site, offers a uniquely preserved archaeological context due to its sudden burial under volcanic ash in 79 AD. This environment presents both exceptional research opportunities and substantial conservation challenges, making it an ideal candidate for the application of robotic and AI-based restoration technologies.

Extensive excavations have revealed public buildings, Roman villas adorned with frescoes, numerous temples—most notably those of Apollo and Isis—fountains, theaters, and the Forum, the city’s central commercial hub [84].

#### 4.2.1. The Use of Robotic Arms

The RePAIR Project (Reconstructing the Past: Artificial Intelligence and Robotics Meet Cultural Heritage) [75,85], launched in 2011, exemplifies the use of robotics and AI in archaeological conservation, specifically for restoring ancient frescoes in Pompeii.

- **Application context:** In Pompeii, thousands of fragmented frescoes had remained unsorted for decades, posing a major challenge for conservators.
- **Description of the technology:** Robotic arms, guided by computer vision and machine learning algorithms, were employed to scan, classify, and autonomously propose matches among fresco fragments.
- **Results of the robotic application:** The hybrid human–machine approach accelerated restoration workflows and minimized physical handling of fragile materials, directly contributing to conservation efforts.
- **Challenges and limitations:** No specific challenges or unresolved issues were detailed.
- **Lessons learned and future impact:** This project demonstrates the value of integrating robotics and AI into heritage restoration, with potential for broader application to large-scale fragment reassembly tasks.

#### 4.2.2. The Use of Robot SPOT

In addition to the aforementioned findings, spaces with limited accessibility and structural instability have been uncovered, posing challenges for exploration due to collapse risks. Pompeii’s unique architecture, including its underground structures, presents difficulties for archaeologists in terms of both access and study. In Pompeii, robots are used as part of a multilevel monitoring strategy to inspect structures and terrain, helping assess damage, material decay, and structural stability, ultimately supporting data-driven decisions for efficient and resilient cultural heritage maintenance [86].

In this context, the SPOT robot developed by Boston Dynamics is used [87].

- **Application context:** SPOT is used in Pompeii to safely and precisely map areas that are difficult to access, such as confined spaces, in order to support the study of antiquities and the planning of restoration interventions.
- **Description of the technology:** SPOT is a quadruped robotic dog developed by Boston Dynamics, weighing approximately 33 kilograms. It is equipped with 360° vision for obstacle avoidance, a LIDAR system for 3D mapping, and two operational modes: the Leica BLKARC sensor and the Spot CAM sensor.
- **Results of the robotic application:** SPOT can collect valuable data from confined areas, monitor the progress of restoration works efficiently, and perform repetitive or time-consuming tasks autonomously and effectively.
- **Challenges and limitations:** Safety conditions inside underground tunnels created by tomb raiders are highly precarious; SPOT is used to explore these safely and speedily.
- **Lessons learned and future impact:** The use of SPOT demonstrates how autonomous robots can enhance archaeological documentation and assist in planning and monitoring interventions.

SPOT also contributes to the security and surveillance of the Pompeii site by conducting routine inspections to identify structural problems, signs of erosion and damage, or vandalism. Its onboard cameras detect movement and patrol visitor-accessible areas, deterring potential looters and inspecting underground tunnels dug by “tombaroli”—looters seeking valuable artifacts in the city’s ruins [88].

### 4.3. Robotic Applications at the Smithsonian Institution

The Smithsonian Institution has been a pioneer in introducing robotics into museum practice, employing different types of robotic systems to support both visitor interaction and collection management. A detailed breakdown of the system is provided below:

- **Context:** The Smithsonian National Museum of American History deployed Pepper [89,90,91,92], a humanoid robot, to enhance the visitor experience, support educators during school visits, and promote engagement among younger and more diverse audiences.
- **Technology Used:** Pepper is a speech-enabled humanoid robot capable of mimicking human expressions, perceiving motion, and interacting with its environment. It includes a touchscreen interface, multilingual support (21 languages), and artificial intelligence that enables it to detect visitor proximity and initiate conversations. It was used to greet visitors, narrate stories, and provide exhibit information.
- **Outcomes:** Pepper attracted first-time visitors, increased repeat attendance, and served as a valuable tool for educators. It particularly appealed to children and tech-savvy audiences, making the museum experience more engaging and accessible.
- **Challenges and Limitations:** Despite its success in drawing attention, Pepper’s interactive abilities were limited to scripted responses and predefined behaviors. Long-term visitor engagement varied depending on age group and content relevance.
- **Lessons Learned and Future Directions:** Humanoid robots like Pepper offer an innovative interface for visitor interaction, but their effectiveness depends on adaptive content, updated narratives, and seamless integration into the overall museum interpretation strategy.

### 4.4. Social Robot in the Archaeological Museum of Thessaloniki

The Archaeological Museum of Thessaloniki is the first cultural institution in Greece to introduce a robotic system as part of its educational and visitor engagement strategy. As a pioneer in the integration of new technologies in cultural spaces, the museum aims to enrich the visitor experience while reinforcing its educational and cultural mission. The system was developed under the innovative program “CultureID: The Internet of Culture – Integrating RFID Technology in the Museum,” which focused on the digital transformation of museum practices. The robot interacts with visitors using pre-programmed dialogues and simple, accessible language. It provides real-time information about the museum’s most significant artifacts, responds to visitor questions, and participates in educational programs such as interactive games for children, including a clue-based riddle game supported by portable RFID devices [93].

A detailed breakdown of the system is provided below:

- **Context:** The robotic system was implemented to promote interactivity, accessibility, and engagement, especially among younger audiences and school groups, in the museum’s permanent exhibitions.
- **Technology Used:** A wheeled mobile robot equipped with RFID integration, navigation sensors, touchscreen interface, and basic speech interaction. It could autonomously navigate the museum space, recognize RFID-tagged exhibits, and interact with users through preset content delivery.
- **Outcomes:** The robot successfully increased visitor engagement, particularly with children, and served as an effective educational assistant. It enhanced the museum’s image as an innovative institution and offered a playful, interactive dimension to the museum experience.
- **Challenges and Limitations:** The robot had limited autonomy and relatively basic natural language capabilities, which constrained its interactivity. Additionally, crowded or dynamic environments occasionally disrupted its navigation performance.
- **Lessons Learned and Future Directions:** This implementation highlights the importance of balancing novelty with long-term functionality. Future developments should focus on more adaptive AI, improved voice interaction, and personalized visitor experiences.

Overall, the case demonstrates the potential of social robotics in heritage education, especially when paired with playful learning strategies and accessible interaction models.

### 4.5. Restoration of Cathedrals—The Case of Notre-Dame de Paris

Notre-Dame de Paris, France, a masterpiece of Gothic architecture constructed in the 12th century, is among the most visited monuments worldwide. The devastating fire of April 2019 caused severe structural damage, including the collapse of the spire and roof. This tragedy accelerated the integration of advanced digital technologies in restoration workflows. High-resolution photogrammetry facilitated the generation of highly detailed 3D digital models of the cathedral [94,95]. This digital reconstruction was compared to pre-fire scans, providing a critical reference framework for monitoring degradation and guiding restoration interventions. A key innovation in the Notre-Dame restoration was also the creation of a digital twin—a detailed virtual replica integrating 3D scans, sensor data, and expert annotations [96].

Regarding the use of robots, the site’s challenging conditions—such as the crowded overhead space, various obstacles (like debris and equipment), blocked GPS signals, low light, and unstable air (e.g., swirling winds)—made the use of drones for surveying very difficult or even impossible [95]. However, for debris removal and other simple operations, robots were utilized. In particular, for example, a remote-controlled robot, originally designed for tunnel inspections, was used to tow wheeled tripods carrying microphones through the cathedral [97]. This setup enabled precise and repeatable acoustic measurements along predefined paths while minimizing human presence in restricted areas.

### 4.6. Use of Robotic Systems in Archaeological Excavation and Documentation—The RASCAL System

During archaeological excavations, thousands of ceramic sherds are uncovered daily, serving as key chronological and cultural indicators. Traditional manual documentation of these fragments is time-consuming, prompting the need for automation. The RASCAL (Robotic Arm for Sherds and Ceramics Automated Locomotion) system, as presented by [24], addresses this challenge through a robotic arm equipped with a camera and multiple data acquisition stations. It operates under varying lighting conditions and can weigh, photograph, and store data for each sherd with high accuracy—processing approximately 1260 fragments per day. The system also supports semi-automated reassembly and 3D modeling of ancient vessels, thereby minimizing physical handling and reducing the risk of damage.

Despite the advantages of robotic integration in cultural heritage documentation—efficiency, precision, and preservation—certain limitations persist. High costs and technical expertise are required for deployment. According to [98], data accessibility is another concern, as 3D datasets are often private and unavailable to the broader research or educational community. Additionally, virtual reality applications that accompany such robotic systems may prioritize technological presentation over archaeological context, potentially diminishing the cultural and educational value of the artifacts themselves.

### 4.7. Discovery of an Ancient Metropolis in the Amazon Jungle Using UAVs and LiDAR

The use of unmanned aerial vehicles (UAVs), particularly when integrated with LiDAR and thermal imaging technologies, has significantly advanced archaeological research in dense tropical environments like the Amazon [99,100,101,102]. Projects such as “Amazônia Revelada” have demonstrated the effectiveness of aerial platforms in mapping previously unknown archaeological sites while preserving natural habitats. These technologies have not only uncovered evidence of complex societies and infrastructures but have also refined ecological monitoring and species identification in regenerating forests.

- **Context:** Archaeologists working in the Amazon rainforest have employed UAVs and LiDAR technology to overcome the challenges posed by dense vegetation, aiming to discover, map, and document hidden archaeological structures non-invasively.
- **Technology Used:** UAVs equipped with LiDAR sensors, thermal imaging, RGB cameras, and GPS/GIS systems. LiDAR pulses penetrated dense canopy layers to produce accurate 3D ground models. Multi-sensor integration allowed for enhanced spatial resolution and detailed analysis.
- **Outcomes:** Discovery of 30 archaeological sites and geometric structures in Brazil; identification of 6000 platforms and urban infrastructure in Ecuador; mapping of roads, canals, and ceremonial spaces dating back 2500 years; enhanced understanding of ancient Amazonian civilizations, challenging previous assumptions about nomadic habitation; application to species-level forest monitoring.
- **Challenges and Limitations:** The non-destructive requirement and the need for canopy penetration suggest technological dependence on specific environmental conditions.
- **Lessons Learned and Future Directions:** UAV-based remote sensing has proven essential for archaeological discovery in rainforest ecosystems. The combination of aerial data, AI-enhanced analysis, and geolocation opens avenues for deeper exploration, urban reconstruction, and conservation of cultural heritage.

To synthesize the analysis of some important case studies presented above in our work, Table 4 outlines a comparative framework that covers the basic technological, archaeological, and operational aspects of each case. This structured approach facilitates a deeper understanding of the benefits and limitations of robotic applications in diverse archaeological contexts.

#### Limited or Unsuccessful Applications

While most applications of robotics in archaeology present impressive and innovative results, there are also instances where the technological implementation did not meet expectations due to technical, environmental, or operational constraints. Critically assessing such cases is essential to draw lessons and form realistic expectations for future implementations.

- **Social robots in museums:** Robots like *Pepper*, although successful in increasing visitor engagement, faced challenges in noisy environments and showed limited acceptance among older adults and less tech-savvy visitors. Moreover, privacy concerns arose due to the embedded cameras and sensors [89].
- **Robotic arms for restoration:** In the RePAIR project [75], despite progress in automatic fragment assembly, failures occurred due to poor preservation conditions or insufficient geometric information [103]. In some cases, AI-based matches lacked proper archaeological justification.
- **UAV operations in tropical environments:** In regions like the Amazon rainforest, dense canopy and high humidity reduced the effectiveness of LiDAR-equipped UAVs. Weather conditions, limited flight autonomy, and the need for complex data post-processing delayed archaeological analysis [35,38].
- **Economic feasibility:** Several robotic initiatives remain experimental or have limited scalability due to high equipment and maintenance costs. The lack of sustainable funding and technical personnel makes long-term deployment in field excavations uncertain.

Although we recognize these constraints, we do not diminish the significance of robotics in archaeology. On the contrary, it contributes to a deeper comprehension of its boundaries and supports better-informed and smarter design and implementation in future solutions and projects.

## 5. Conclusions: Scientific Challenges, Limitations, and Future Directions of Robotics in Archaeology

The integration of robotic technology offers numerous possibilities and opportunities in the field of archaeology. Nevertheless, there are several challenges and limitations that arise in the processes of discovery, documentation, and preservation of archaeological findings. These technologies bring with them a range of constraints that must be taken into consideration.

### 5.1. Technological Limitations

Despite the progress made in robotics, robots face significant difficulties when operating in irregular and unpredictable environments such as archaeological sites. Navigation across uneven terrain requires advanced sensor systems and algorithms for obstacle avoidance and the execution of delicate tasks. However, such systems are not always available or reliable. One fundamental limitation is that robots must possess sufficient degrees of freedom to achieve high flexibility and successful navigation. As such, path planning must involve feasible trajectories that help avoid unexpected situations [104].

### 5.2. Economic Limitations

Robotic systems used in archaeology represent a highly innovative practice that combines technological advancement with the need for careful and detailed study of cultural heritage. However, these systems are expensive to develop and maintain. Despite their advantages, the main challenge remains their high initial purchase and installation costs, especially in archaeological projects or small research institutions with limited budgets. Moreover, the investment in robotics requires specialized personnel, and the cost of their training adds further financial burden, increasing operational costs and expenses. Due to their high cost, robotic systems are produced in limited quantities, forcing institutions to evaluate their cost-effectiveness, which casts doubt on their economic viability [105]. Thus, strategies must be explored that promote funding tools and support innovation in the field of archaeology.

### 5.3. Adaptability Limitations and Integration Challenges

The integration of robotics in archaeology requires effective interdisciplinary collaboration among archaeologists, computer scientists, and engineers, each of whom contributes essential knowledge and expertise. Effective communication channels are crucial to combine skills from each scientific field and ensure the successful implementation of robotic technologies. Archaeologists provide interpretation and evaluation of findings, computer scientists contribute to data processing and analysis, and engineers focus on developing and adapting robotic technologies for archaeological use [106]. However, differences in priorities and misunderstandings between disciplines can undermine collaboration and reduce its effectiveness. Additionally, each archaeological site is unique, with specific environmental and contextual conditions, meaning robotic systems must offer customized solutions. This increases both the cost and the technical complexity of deployment.

### 5.4. Ethical and Social Limitations

While robotics offers new possibilities for more discovery, documentation, and conservation, it also prompts important ethical and social challenges. A significant concern centers on substituting human labor, leading to the decline of conventional archaeological skills. Automation may reduce the human role in excavation and data collection, weakening experiences and potentially isolating communities from their traditions [107]. Furthermore, the use of robotics in sensitive or important cultural environments must be handled with careful planning and, of course, respect for the legacy [108]. Maintaining the original condition of archaeological artifacts and honoring the cultural and spiritual significance of specific sites remain crucial. Another pressing issue is the ownership and regulation of data obtained through robotic methods, particularly via UAVs or AUV vehicles. When working across borders or within indigenous territories, complex questions are raised about the ownership of generated 3D models and datasets, which becomes a contentious issue, particularly in indigenous or international heritage settings. Emerging ethical frameworks call for stronger recognition of data sovereignty and local stakeholder involvement in decisions around storage, access, and reuse [109].

Addressing these issues is not only significant to the development of robotic archaeology but key to its responsible incorporation within heritage management. Ensuring social sensitivity and inclusivity is essential in future technological developments.

### 5.5. Future Directions

Future developments in robotic archaeology should focus on making systems more intelligent, modular, and responsive to complex environments. Advances in AI—such as more robust learning models and enhanced decision-making capabilities—can enable robots to operate with greater autonomy and contextual awareness. The integration of advanced sensors, including multispectral and 3D imaging, will further improve data quality and interpretation. In museum settings, social robots could become more engaging through improved speech recognition, interaction design, and personalized visitor experiences. On-board processing using GPUs can also support these capabilities by enabling faster, real-time analysis.

## Figures and Tables

**Table 1:** Overview of robotic systems, their functionalities, and archaeological applications.

Robot Type	Core Functions	Key Technologies	Archaeological Applications
Aerial Robots (UAVs) [21,22]	Aerial mapping, scanning, documentation	LiDAR, photogrammetry, thermal imaging	Landscape archaeology, site detection, topographic mapping, monitoring inaccessible areas
Underwater Robots (AUVs, ROVs) [18,19]	Exploration, scanning, imaging	Sonar, stereo vision, GPS–acoustic, manipulators	Shipwreck surveys, submerged architecture, seafloor mapping, deep-sea excavation
Ground Robots (UGVs) [20]	Mobility on rough terrain, data acquisition	Cameras, IMUs, LiDAR, terrain navigation algorithms	Inspection of fragile ruins, tunnel exploration, mapping of collapsed structures
Manipulative Robots [23,24]	Object handling, fragment reconstruction	Robotic arms, AI-based matching, 3D vision	Artifact assembly, restoration support, conservation labs
Social Robots [25,26]	Visitor interaction, education	Speech recognition, touchscreens, movement tracking	Museum tours, interactive education, accessibility support

**Table 2:** Comparative summary of underwater robotic sensors, including purpose, application scenarios, accuracy, and cost indicators.

Sensor Type	Purpose	Application Scenarios	Accuracy Indicators	Cost Range
Stereo Vision [53,58], Cameras	Visual documentation, 3D modeling	Shallow water, museum setups, confined spaces	Resolution up to 0.1 mm	Low–Medium
GPS–Acoustic [18]	Underwater localization	AUV/ROV missions in open water, deep dives	±1–2 m	High
Lighting	Illumination in dark/deep environments	Caves, shipwreck interiors, deep zones	N/A	Low
Sonar, Multibeam [52,54]	Seafloor mapping, object detection	Deep sea, low-visibility zones, turbid waters	0.5 m horizontal accuracy	High
Inertial Measurement Units (IMUs) [54,58]	Motion and orientation tracking	Navigation, pose estimation, confined operations	±0.1–0.5°	Medium
Depth Sensors [55]	Depth control	Submerged archaeological sites, stratified layers	±0.1 m	Low
Force/Manipulator Sensors [58]	Artifact interaction and manipulation	Robotic arms handling fragile or embedded objects	<1 N sensitivity	Medium–High
Magnetometers [18]	Detect metal artifacts	Metal tool detection, shipwrecks, hidden chambers	Detects ferrous at 1–3 m	Medium
Environmental Sensors [56]	Site preservation assessment	Long-term monitoring of sealed tombs or crypts	±0.5 °C/±3% RH	Low

**Table 3:** Special features of robotic applications in archaeological contexts.

Application	Special Features/Differentiators
Exploration of the Great Pyramid of Giza, Egypt	High-resolution cameras for low-light conditions Navigation in narrow passages
Underwater exploration of the submarine volcano Kolumbo (Santorini)	Accurate mission area mapping 3D model generation
Mapping and monitoring archaeological sites (e.g., Pompeii)	High-precision sensor data collection Access to hazardous or hard-to-reach areas Site preservation support
Smithsonian National Museum of American History	Interactive information and tours Creative user interaction
Archaeological Museum of Thessaloniki	Participates in treasure hunt games for children Provides museum information via portable devices Educational and entertainment goals
San Antolín Cathedral (Palencia, Spain)	3D modeling via laser scanning and photogrammetry Environmental condition monitoring Damage assessment tools
Analysis of ancient ceramics from excavations	Digital archiving and classification Comparison with known collections Faster, safer ceramic analysis
Mapping the archaeological site of Wombwell Wood	Non-invasive structure detection in dense vegetation Fast, large-area, high-resolution 3D mapping GIS integration
Archaeological mapping in the Amazon Jungle	Dense jungle penetration using LiDAR AI-powered autonomous UAV navigation Sensor fusion (thermal, GIS) for 3D reconstruction Minimal environmental impact

**Table 4:** Comparative framework of selected case studies in archaeological robotics.

Case Study	Technologies	Challenges	Solutions	Results/Impact	Limitations/Issues
Djedi Project—Giza, Egypt	Micro-robot, snake camera, drill, sensors	Access to narrow, confined shafts	Remote exploration with minimal impact	Discovery of hidden corridors, structural imaging	Very limited space, high design complexity
Pompeii—Italy	SPOT robot, LiDAR	Fragile areas, instability, dispersed fragments	Terrain inspection, robotic reassembly	Monitoring, data collection, restoration	High cost, technical expertise required
Smithsonian Museum—USA	Social robot, speech, sensors	Enhance visitor engagement	Interactive, multilingual guidance	Improved accessibility	Privacy concerns, limited acceptance
Amazon Rainforest	UAVs with LiDAR and thermal sensors	Dense vegetation, inaccessibility	Non-invasive aerial mapping	Discovery of hidden settlements	Weather sensitivity, complex processing
